# A stem-less probe using spontaneous pairing between Cy3 and quencher for RNA detection

**DOI:** 10.1080/14686996.2016.1182412

**Published:** 2016-07-05

**Authors:** Hiromu Kashida, Kazuhiro Morimoto, Hiroyuki Asanuma

**Affiliations:** ^a^ Graduate School of Engineering, Nagoya University, Furo-cho, Chikusa-ku, Nagoya464-8603, Japan; ^b^ Japan Science and Technology Agency, PRESTO, 4-1-8 Honcho, Kawaguchi, Saitama332-0012, Japan

**Keywords:** DNA, Cy3, RNA imaging, Fluorescence in situ hybridization, 30 Bio-inspired and biomedical materials, 101 Self-assembly/Self-organized materials, 208 Sensors and actuators, 301 Chemical syntheses/processing, 501 Chemical analyses, 505 Optical/Molecular spectroscopy

## Abstract

We herein report a stem-less probe for the detection of RNA that depends on pairing between Cy3 and nitro methyl red. In our design, two Cy3 residues and two nitro methyl red residues were introduced into an oligonucleotide. In the absence of the target, these dyes formed a complex, and emission of Cy3 was efficiently quenched. Hybridization with the target RNA disrupted this interaction and resulted in Cy3 emission. Under optimized conditions, the signal to background ratio was as high as 180. We demonstrated specific detection of target RNA in cells using a wash-free FISH protocol.

## Graphical abstract

**Figure:**
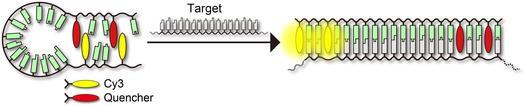

## 1. Introduction

Fluorescent oligonucleotide probes are essential tools in biology, biotechnology, and nano-medicine research. Ideally, a probe should emit a light signal when hybridized to the target. Molecular beacon probes are oligonucleotides with a fluorophore at one end and a quencher at the other.[1] Formation of a stem-loop structure in the absence of a target brings the fluorophore and quencher into close proximity and results in quenching of fluorescence. When the probe is hybridized to target DNA or RNA, a signal is observed. Molecular beacons have been widely used to detect DNA and RNA;[2,3] however, the stable base pairing of the stem portion of the molecular beacon may result in slow response to target. Linear probes that do not depend on stem-loop structures for quenching have been reported by several groups.[4–12]

We have reported that dyes incorporated into oligonucleotides through a D-threoninol linker can act as surrogate “base pairs” to stabilize DNA duplexes.[13] Recently, we reported that pyrene and anthraquinone incorporated through the D-threoninol linker act as hetero-selective pseudo base pairs.[14] These pairs are so stable that pyrene oligomers and anthraquinone oligomers form a DNA-like duplex. These studies indicated that a fluorophore and a quencher can form a complex even in the absence of natural base pairs. We reasoned that self-quenching complexes would be useful in stem-less probes.

**Figure 1.**
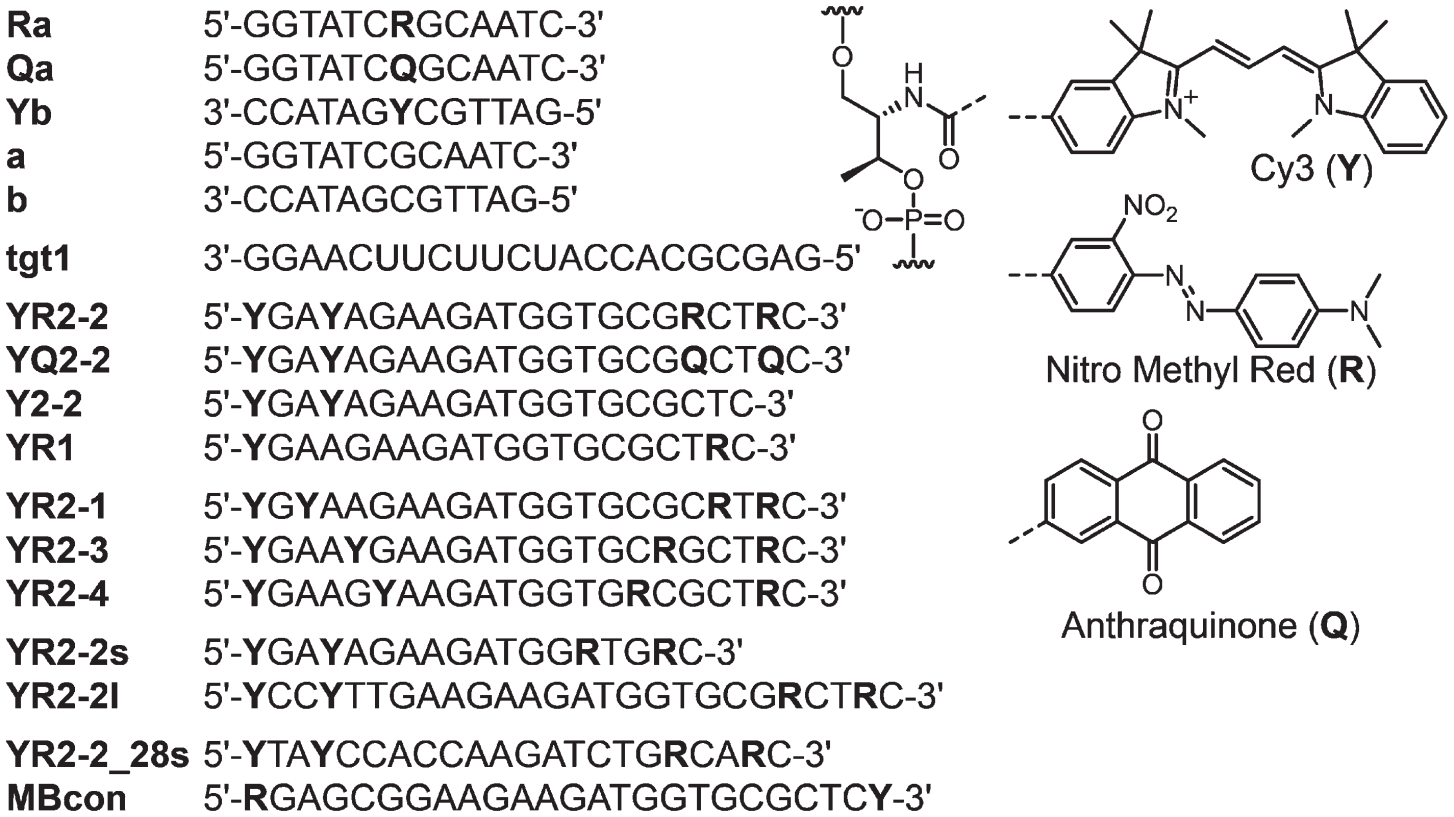
Sequences of oligonucleotides used in this study. Chemical structures of dyes and D-threoninol linker used to incorporate dyes into oligonucleotides are shown.

**Figure 2.**
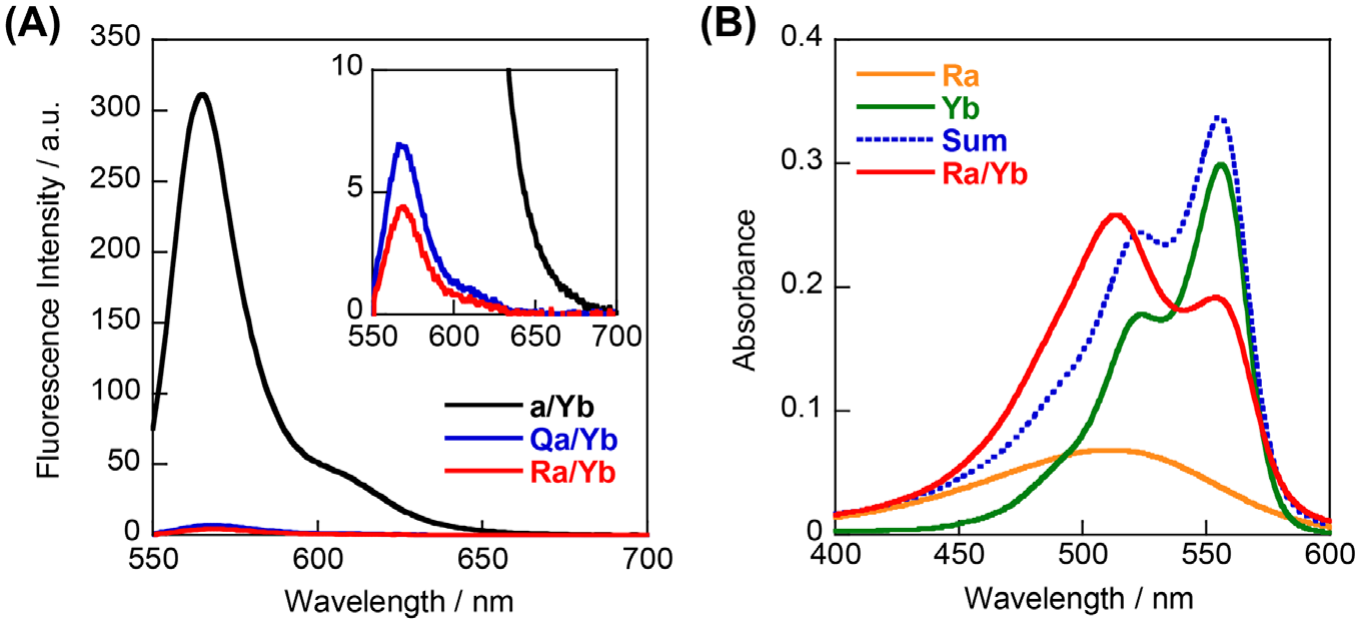
(A) Fluorescence spectra of model duplexes **a/Yb**, **Qa/Yb**, and **Ra/Yb**. Conditions: 2.0 μM quencher strands and 1.0 μM **Yb** in 100 mM NaCl, 10 mM phosphate buffer (pH 7.0), 20 °C. (B) UV-visible absorption spectra of single-strands **Ra** and **Yb**, and **Ra**/**Yb** duplex. Summation of spectra of **Ra** and **Yb** is shown in blue dotted line. Conditions: 2.0 μM each strand, 100 mM NaCl, 10 mM phosphate buffer (pH 7.0), 20 °C.

**Figure 3.**
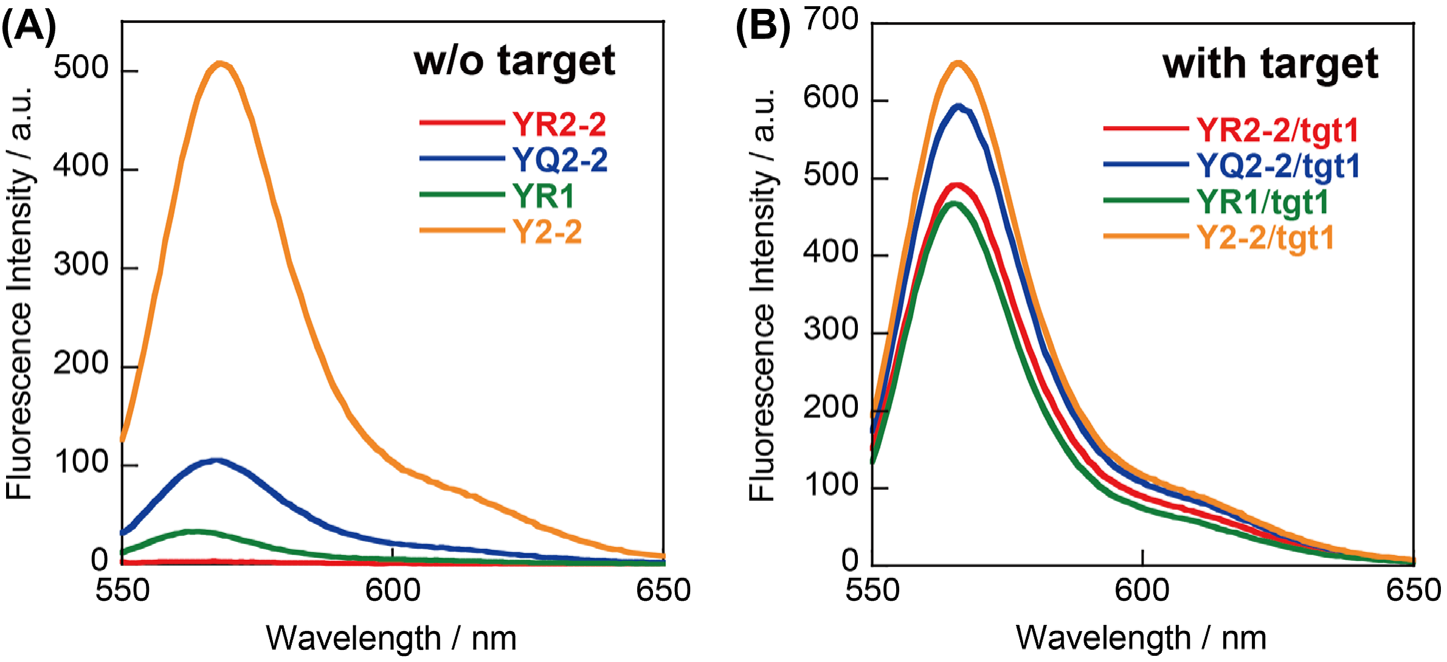
Emission spectra (A) without and (B) with RNA target of probes **YR2–2**, **YQ2–2**, **YR1**, and **Y2–2**. Conditions: 0.2 μM probe, 0.4 μM **tgt1**, 100 mM NaCl, 10 mM phosphate buffer (pH 7.0), 20 °C.

**Figure 4.**
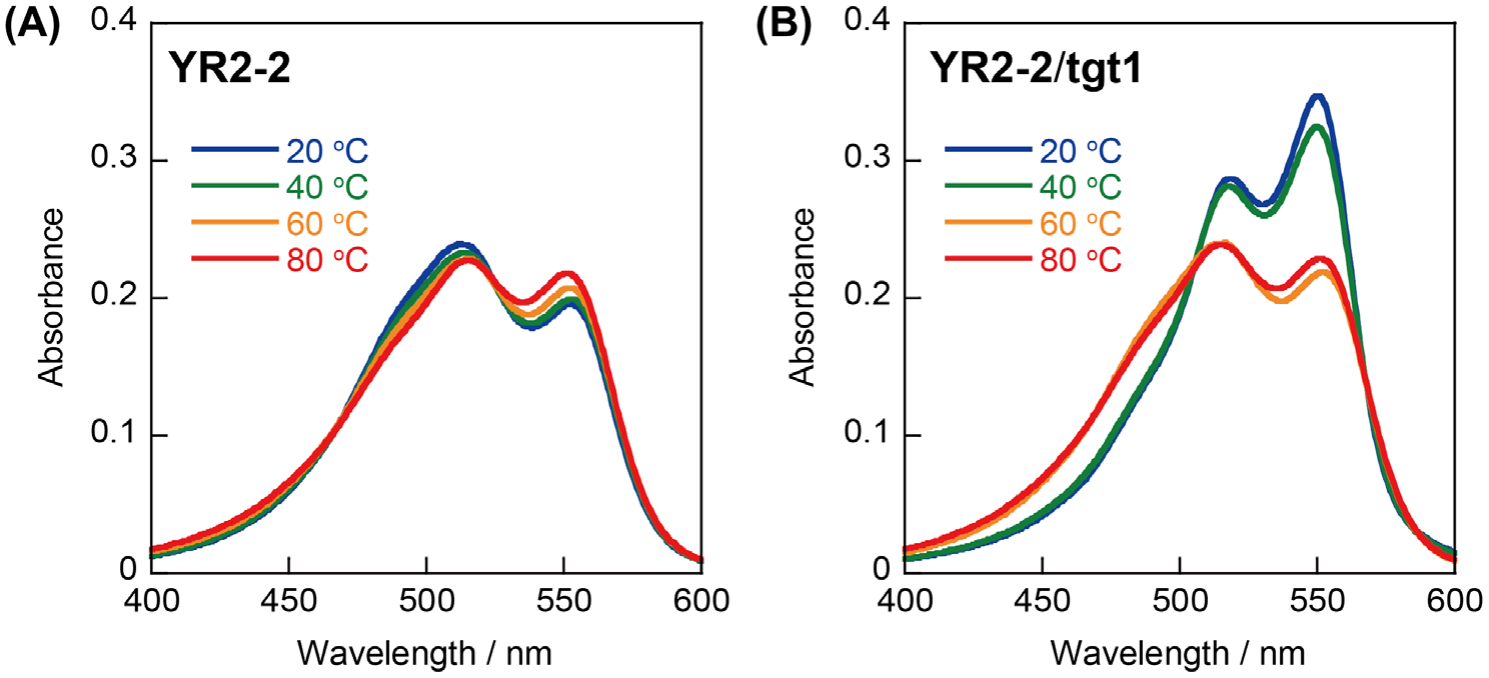
Effects of temperature on UV-visible absorption spectra of **YR2–2** (A) without or (B) with target RNA. Conditions: 1.0 μM each strand, 100 mM NaCl, 10 mM phosphate buffer (pH 7.0).

**Figure 5.**
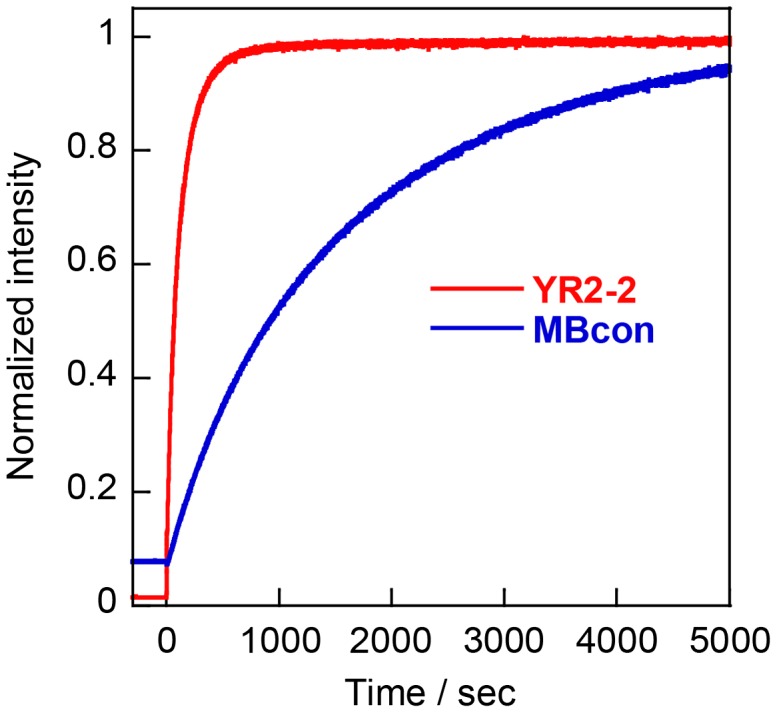
Responses of **YR2–2** and conventional molecular beacon (**MBcon**) to the target (**tgt1**). Target RNA was added at the point of 0 s. Conditions: 0.2 μM probe strands, 0.4 μM **tgt1**, 100 mM NaCl, 10 mM phosphate buffer (pH 7.0), 20 °C.

**Figure 6.**
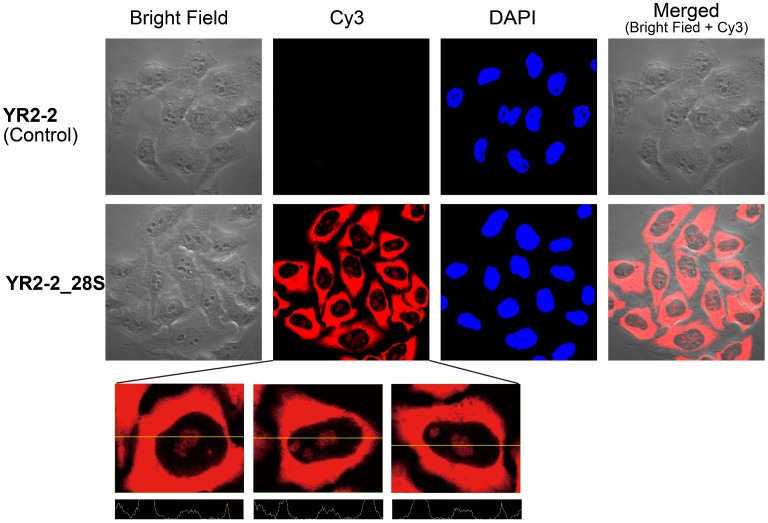
Confocal microscopy images of fixed and permeabilized HeLa cells treated with **YR2–2** or **YR2–2_28S** without washing prior to imaging. Nuclei were stained with DAPI. Intensity analyses of cells are shown at the bottom.

**Scheme 1.**
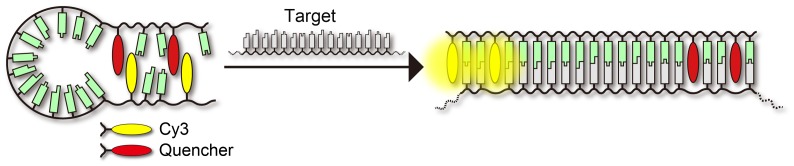
Schematic illustration of our strategy to detect RNA by using a stem-less probe.

Previously we reported stem-less probes that depend on perylene as a fluorophore.[15,16] These probes have multiple perylene residues, and emission from perylene is quenched through non-emissive complex formation between perylene residues. This strategy cannot be applied to other fluorophores such as Cy3 due to their low self-quenching efficiencies. Herein we report a new probe design based on spontaneous complex formation between Cy3 and a quencher. Our strategy is shown in Scheme 1. Cy3 and various quencher residues were introduced near either end of a linear oligonucleotide. Unlike a conventional molecular beacon probe, this oligonucleotide does not have a self-complementary sequence. Nevertheless, Cy3 and the quencher spontaneously formed a complex in the absence of the target so that Cy3 emission was efficiently quenched. Upon duplex formation with a complementary target, strong Cy3 emission was observed. Because this probe does not have a stem-loop structure, a high response speed in the presence of a target was expected. Here, we report quenching efficiencies and stabilities of complexes between Cy3 and a quencher in the context of model duplexes. Detection using the stem-less probes was optimized *in vitro*, and detection of 28S rRNA in cells without washing procedures was demonstrated.

## 2. Experimental details

### 2.1. General

All conventional phosphoramidite monomers, controlled-pore glass columns, and reagents for DNA synthesis were purchased from Glen Research (Sterling, VA). Other reagents for the syntheses of phosphoramidite monomers were purchased from Tokyo Chemical Industry (Tokyo, Japan), Wako (Osaka, Japan), or Aldrich (St. Louis, MO). Unmodified oligonucleotides were purchased from Integrated DNA Technologies (Coraville, IA).

All the modified oligodeoxyribonucleotides (ODNs) were synthesized on an automated DNA synthesizer (M-6-MX, Nihon Techno Service Co., Ltd, Tsukuba, Japan) using phosphoramidite monomers bearing Cy3 (**Y**), 4-dimethylamino-2′-nitroazobenzene (nitro methyl red; **R**), and anthraquinone (**Q**) residues. Syntheses of phosphoramidite monomers were reported previously.[17,18] The scheme for synthesis of the phosphoramidite monomer tethering *p*-nitroazobenzene is shown in Supporting Information. ODNs were purified by reversed phase HPLC and characterized using a matrix-assisted laser desorption ionization time-of-flight mass spectrometer (MALDI-TOF MS; Autoflex, Bruker Daltonics, Bremen, Germany). MALDI-TOF MS data for the synthesized ODNs (Figure 1): **Ra**, observed 4109 (calculated for [**Ra** + H^+^], 4108); **Qa**, observed 4047 (calculated for [**Qa** + H^+^], 4046); **Yb**, observed 4194 (calculated for [**Yb** + H^+^], 4195); **YR2–2**, observed 7610 (calculated for [**YR2–2** + H^+^], 7614); **YQ2–2**, observed 7487 (calculated for [**YQ2–2** + H^+^], 7490); **Y2–2**, observed 6683 (calculated for [**Y2–2** + H^+^], 6688); **YR1**, observed 6601 (calculated for [**YR1** + H^+^], 6600); **YR2–1**, observed 7593 (calculated for [**YR2–1** + H^+^], 7614); **YR2–3**, observed 7586 (calculated for [**YR2–3** + H^+^],7614); **YR2–4**, observed 7590 (calculated for [**YR2–4** + H^+^], 7614); **YR2–2s**, observed 6385 (calculated for [**YR2–2s** + H^+^], 6403); **YR2–2 l**, observed 8770 (calculated for [**YR2–2 l** + H^+^], 8800); **YR2–2_28s**, observed 7436 (calculated for [**YR2–2_28s** + H^+^], 7438).

### 2.2. Spectroscopic measurements

Fluorescence spectra were measured on an FP-6500 spectrometer (JASCO, Tokyo, Japan) equipped with a microcell holder. Excitation wavelength was 546 nm. UV-visible absorption spectra were measured on a UV-1800 spectrometer (Shimadzu, Kyoto, Japan) equipped with a programmable temperature controller; 10-mm quartz cells were used. The melting curves were recorded by measuring the change in absorbance at 260 nm versus temperature. The melting temperature (*T*_m_) was determined from the maximum in the first derivative of the melting curve. Both the heating and the cooling curves were measured, and the calculated *T*_m_ values from these curves agreed to within 2.0 °C. The temperature ramp was 0.5 °C min^−1^.

### 2.3. Cell culture and fluorescence *in situ* hybridization (FISH)

HeLa cells were cultured in Dulbecco’s modified Eagle’s medium (DMEM) supplemented with 10% fetal bovine serum, 80 μg ml^–1^ penicillin, and 90 μg ml^–1^ streptomycin on a cover glass placed in the bottom of a well of a 12-well plate. Cells were cultured at 37 °C with 5% CO_2_ in humidified air. Cells were fixed in phosphate-buffered saline (PBS) containing 4% paraformaldehyde at room temperature for 30 min. HeLa cells were treated with PBS containing 0.2% Triton-X100, 10 mM glycine, and 0.01% NaN_3_ for 5 min, and then 1.6 μM probe was applied to cells at room temperature for 1 h. Excess probe was removed by rinsing twice with PBS containing 10 mM glycine, and 0.01% NaN_3_. This washing procedure was omitted in wash-free FISH experiments. Cells were embedded in Mowiol (Calbiochem, San Diego, CA) prior to imaging. The stained HeLa cells were visualized using FV-1000 confocal laser microscopy (Olympus, Tokyo, Japan). Images were taken with a 100× oil emission objective lens. The 543 nm laser was used to excite the Cy3 with emission collected using a 555–655 nm band path filter.

## 3. Results and discussion

### 3.1. Evaluation of quenching efficiency using model duplexes

We first evaluated efficiencies of quenching of Cy3 (**Y**) in the context of model oligonucleotides by nitro methyl red (**R**) and anthraquinone (**Q**), both known to efficiently quench fluorophores.[19,20] Model 13-mer duplexes with **Y**-**R** or **Y**-**Q** pairs (**Ra/Yb** or **Qa/Yb**, Figure 1) were prepared. Emission spectra of **Ra/Yb** and **Qa/Yb** duplexes are shown in Figure 2(A). Both **R** and **Q** residues strongly quenched the emission of Cy3 compared with a duplex without a quencher (**a**/**Yb**). The quenching efficiency of the **R** residue was slightly higher than that of the **Q** residue.

Hybridization of **Yb** with **Ra** induced remarkable hypochromicity and hypsochromicity of the bands at around 550 and 515 nm, respectively, compared with a summation spectrum of single-stranded **Ra** and **Yb** (compare red line with blue dotted line in Figure 2(B)). These changes are characteristic of hetero H aggregates. The ratio of absorbance at 550 nm to that at 515 nm, A_550_/A_515_, of the **Ra**/**Yb** duplex was 0.73, whereas that of the summation spectrum was 1.42. Thus, complex formation between Cy3 and nitro methyl red can be monitored from changes in absorption bands. In contrast, **Qa**/**Yb** exhibited only slight changes in absorption bands, probably due to large difference of absorption bands between Cy3 and anthraquinone (Figure S1).

We estimated the stability of the dye dimers by measuring melting temperatures (*T*_m_) of duplexes (Table 1). The **Ra**/**b** duplex, a duplex with a single **R** residue and no Cy3, was 40.2 °C whereas that of a **Qa**/**b** duplex was 53.3 °C. This indicated that anthraquinone stabilizes a DNA duplex more than nitro methyl red does. This relationship was reversed when a Cy3 residue was introduced opposite the **R** or **Q** residue: **Ra**/**Yb**, the duplex with the **R**-**Y** pair, had a *T*_m_ of 48.2 °C, significantly higher than that of the **Qa**/**Yb** duplex, which was 45.7 °C. Thus, interaction between **R** and **Y** residues was much stronger than that between **Q** and **Y** residues. This stabilization is likely due to the donor–acceptor interaction between Cy3 and nitro methyl red.

**Table 1.** Spectroscopic and thermal properties of a model duplex that contains a pair between Cy3 and a quencher.

Duplex	Emission intensity^a^	S/B ratio^b^	A_550_/A_515_^c^	*T*_m_/˚C ^c^	Δ*T*_m_/˚C ^d^
**Ra/Yb**	3.9	80	0.73	48.2	+8.0
**Qa/Yb**	6.5	48	1.64	45.7	−7.6

^a^ Emission intensity at 565 nm in arbitrary units. Conditions were 2.0 μM **Ra** or **Qa**, 1.0 μM **Yb**, 100 mM NaCl, 10 mM phosphate buffer (pH 7.0), 20 °C.

^b^ Ratio of emission intensity of **a**/**Yb** to **Ra**/**Yb** or of **a**/**Yb** to **Qa**/**Yb**..

^c^ Conditions were 2.0 μM DNA, 100 mM NaCl, 10 mM phosphate buffer (pH 7.0), 20 °C.

^d^ Difference in *T*_m_ between **Ra/Yb** and **Ra**/**b** or **Qa/Yb** and **Qa**/**b**.

To evaluate how the electron density of the dyes impacts the duplex stability, we also measured *T*_m_s of pairs between Cy3 and two other azo compounds, nitroazobenzene and methyl red. A stronger stabilization of the duplex was observed when the electron-rich azo compounds methyl red and nitro methyl red were introduced into the counter position of Cy3 than when anthraquinone or nitroazobenzene were opposite the Cy3 (Table S1). This strongly supports our hypothesis that a donor–acceptor interaction strongly influences the stability of the dye cluster.

### 3.2. RNA detection using stem-less probes

We next synthesized stem-less probes containing two Cy3 moieties and two quenchers (illustrated in Scheme 1, sequences given in Figure 1). Nitro methyl red (**R**) or anthraquinone (**Q**) residues were incorporated as quenchers since these molecules showed high quenching efficiencies in model duplexes. First, 22-mer stem-less probes **YR2–2** and **YQ2–2** were investigated. A control probe without quenchers (**Y2–2**) was also examined. In the absence of the target, background emission of **YR2–2** was much lower than that of **YQ2–2** (Figure 3(A)); this is correlated with the higher stability of the **Y**-**R** pair compared with the **Y**-**Q** pair in model duplexes. The A_550_/A_515_ ratio of **YR2–2** (0.81) was lower than that of **YQ2–2** (1.30), supporting the hypothesis that **Y** and **R** residues form a complex in the ground state at 20 °C (Figure 4).

Emission spectra of **YR2–2** and **YQ2–2** in the presence of target RNA **tgt1** are shown in Figure 3(B). In the presence of target, the A_550_/A_515_ ratio of **YR2–2** increased to 1.23, demonstrating the dissociation of the **Y**-**R** complex. **YR2–2** had slightly lower emission in the presence of **tgt1** than did **YQ2–2**, probably due to FRET from Cy3 to nitro methyl red.[21] The signal to background (S/B) ratio (the ratio of emission in the presence to that in the absence of target) of **YR2–2** was 180 at 20 °C, whereas that of **YQ2–2** was only 5.7 (Table 2). **YR1** that tethers only a single **Y**-**R** pair showed much higher background emission in the absence of the **tgt1** than did **YR2–2** (Figure 3), probably due to insufficient complex formation between **Y** and **R** residues (A_515_/A_550_ was 1.02). The S/B ratio of **YR1** was significantly lower than that of **YR2–2** (Table 2). Thus, two **R** residues and two **Y** residues are necessary for sufficient signal to background, and the stem-less probe containing the two **Y/R** pairs detects target RNA with high sensitivity.

**Table 2.** Detection abilities and duplex stabilities of probes synthesized in this study.

Sequence	Fluorescence intensity ^a^		S/B ratio	A_550_/A_515_^b^		*T*_m_/˚C ^b^
	w/o **tgt1**	with **tgt1**		w/o **tgt1**	with **tgt1**	
**YR2–2**	2.73	491	180	0.81	1.23	51.9
**YQ2–2**	103	589	5.7	1.30	1.41	61.1
**Y2–2**	486	646	1.3	1.33	1.41	61.2
**YR1**	32.6	467	14	1.02	1.45	61.7
**YR2–1**	4.61	260	56	0.80	0.97	55.9
**YR2–3**	9.54	487	51	0.86	1.32	56.6
**YR2–4**	4.20	343	82	0.85	1.39	52.2
**YR2–2s**	3.96	97.6	25	0.82	1.05	28.8
**YR2–2 l**	24.9	358	14	0.95	1.29	57.0

^a^ Emission intensity at 565 nm in arbitrary units. Conditions were 0.2 μM probe, 0.4 μM **tgt1**, 100 mM NaCl, 10 mM phosphate buffer (pH 7.0), 20 °C.

^b^ Conditions were 1.0 μM DNA, 100 mM NaCl, 10 mM phosphate buffer (pH 7.0), 20 °C.

We then optimized the number of bases between Cy3 moieties. Emission spectra of examined probes in the presence and absence of target are shown in Figure S3, and emission intensities are summarized in Table 2. When a single base was inserted between **Y** residues (**YR2–1**), emission intensity in the presence of target decreased relative to that observed with **YR2–2** because of self-quenching between two Cy3 moieties. Emission intensity of **YR2–4**, which has four bases between Cy3 moieties, in the presence of **tgt1** was also lower than that of **YR2–2**. The lower emission of **YR2–4** was probably due to the FRET from Cy3 to nitro methyl red, since Cy3 was only nine bases apart from the quencher in the **YR2–4**/**tgt1** duplex. The emission of **YR2–3**, with three bases between Cy3 residues, in the absence of target was high. Consequently, the highest S/B ratio was attained when two bases were inserted between the Cy3 moieties.

Next, we optimized the length of the probe by varying the number of nucleotides; emission spectra of the probes ranging in length from 18 to 26 nucleotides are shown in Figure S4. When the chain length was 26 residues (**YR2–2 l**), background emission was higher than that of the 22-mer **YR2–2**. Because of relatively high A_550_/A_515_ ratio (0.95), **Y**-**R** complexation appeared to be suppressed in the context of the longer chain (Table 2). In contrast, a shorter probe, the 18-mer **YR2–2s** showed less emission than **YR2–2** in the presence of **tgt1**. This low emission was mainly caused by the low *T*_m_ of the **YR2–2s**/**tgt1** duplex (28.8 °C). From these results, we concluded that a 22-mer probe with two Cy3 moieties separated by two bases is the most efficient probe for RNA detection.

In order to compare the self-quenching probe to a conventional molecular beacon, we compared response speed of **YR2–2** with that of a shared-stem molecular beacon with **R** and **Y** on the termini (**MBcon**). The emission intensity of **YR2–2** reached a plateau within 500 s after the addition of target RNA, whereas the molecular beacon signal did not plateau for over 5000 s (Figure 5). We also evaluated the affinity of **YR2–2** for mismatched targets and found that the emission intensity in the presence of an RNA target with a single mismatch was only one tenth that for the fully matched target (Table S2). In addition, almost no emission was observed in the presence of an RNA with two bases mismatched to the probe. Thus, our self-quenching probe has better response speed than a conventional molecular beacon and high specificity.

### 3.3. RNA detection in cells

Finally, we evaluated the ability of our probe to detect RNA in cells. We designed a probe targeting a region of 28S rRNA (**YR2–2_28S**). The precursor of mature 28S rRNA is processed in the nucleolus and incorporation into the 60S ribosomal subunit follows.[22] Hence, if **YR2–2_28S** specifically detects 28S rRNA, bright emission should be observed in the nucleolus and in the cytoplasm. Our *in vitro* experiments demonstrated that the probe emits light only in the presence of target RNA, which should allow the use of a wash-free FISH protocol. **YR2–2_28S** was added to HeLa cells fixed and permeabilized with Triton X, and fluorescence images were taken with confocal microscopy with and without washing procedure. **YR2–2** was used as a control since no target for this probe should be present in HeLa cells. As expected, almost no emission was detected in fixed HeLa cells treated with **YR2–2** (Figure 6). **YR2–2_28S** afforded strong emission from Cy3 in the cytoplasm as shown by comparison with 4′,6-diamidino-2-phenylindole (DAPI) stained cells. In addition, spatial analysis revealed distinct emission due to **YR2–2_28S** from nucleoli (Figure 6 bottom). Similar localization of Cy3 emission was observed when a conventional FISH protocol involving washing procedures was used (Figure S5). The emission observed using probe **YR2–2_28S** coincided with the previous studies employing 28S rRNA-specific probes;[12,23,24] therefore, we concluded that the self-quenching probe detects target RNA in cells.

## 4. Conclusions

We successfully prepared an oligonucleotide probe that relies on spontaneous pairing between Cy3 and nitro methyl red to quench emission in the absence of target. Melting analyses of a model duplex tethering a Cy3-quencher pair showed that this dimer has a high stability. From results *in vitro*, we concluded that a 22-mer probe, which has two Cy3 and two nitro methyl red residues separated by two natural bases, detected RNA with high efficiency. This optimized probe design enabled sensitive detection of 28S rRNA in HeLa cells using a wash-free FISH protocol. Moreover, since quenching of Cy3 by nitro methyl red occurred spontaneously without the assistance of base pairing, this strategy will be applicable to the design of peptide-based probes.

## Disclosure statement

No potential conflict of interest was reported by the authors.
